# New mechanistic insights into modifiable risk factors that promote pancreatic cancer

**DOI:** 10.18632/oncotarget.26813

**Published:** 2019-03-29

**Authors:** Supriya Srinivasan, Austin R. Dosch, Nagaraj S. Nagathihalli

**Affiliations:** Nagaraj S. Nagathihalli: Sylvester Comprehensive Cancer Center, University of Miami Miller School of Medicine, Miami, Florida, USA; Division of Surgical Oncology, Department of Surgery, University of Miami Miller School of Medicine, Miami, Florida, USA

**Keywords:** CREB, pancreatic cancer, modifiable risk factors, alcohol, smoking

**Figure 1:**
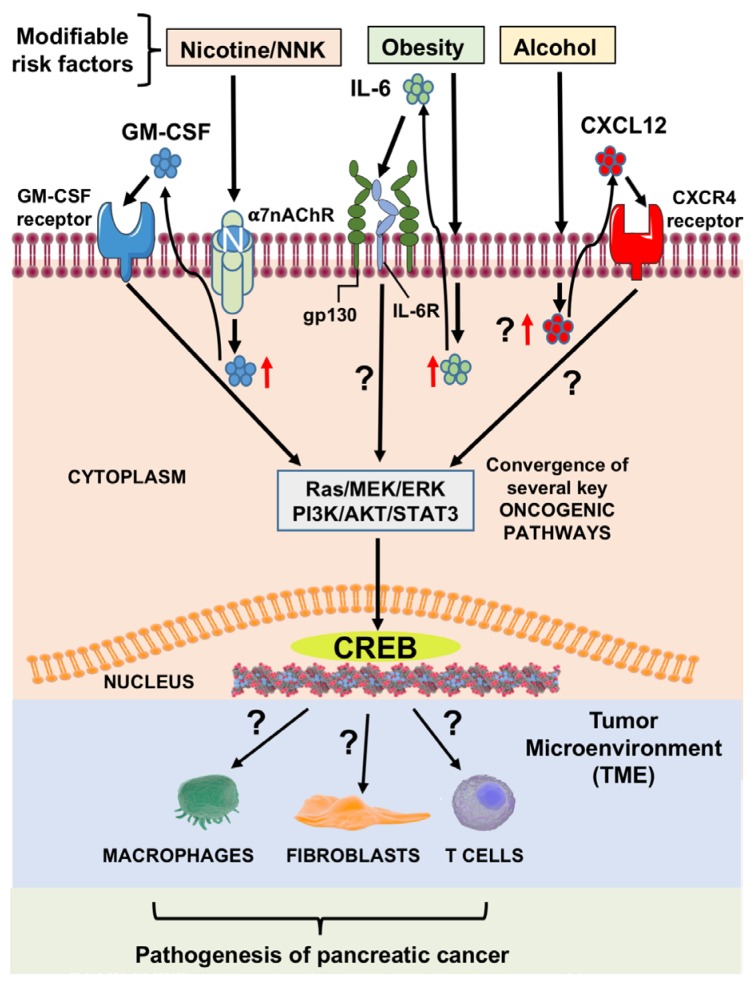
Effect of modifiable risk factors including smoking, obesity and heavy alcohol consumption on pancreatic cancer initiation and progression Molecular mechanisms of modifiable risk factor mediated induction of cytokines involving subsequent activation of CREB, resulting in pancreatic cancer growth and progression. NNK induces GM-CSF release from pancreas cells, which can then activate CREB through AKT/PI3K pathway. Molecular pathway of heavy alcohol-induced CXCL12 release can activate CREB, through CXCR4 pathway (unpublished data). Obesity can activate CREB and lead to STAT3 activation through enhanced IL-6 release. These risk factors alone, or in combination with other factors, can mediate pathogenesis of pancreatic cancer through CREB signaling.

Pancreatic ductal adenocarcinoma (PDAC) carries a poor prognosis and is expected to become the second leading cause of cancer mortality in the United States by 2030 [1]. The dismal outcomes in PDAC are largely attributed to the poor response of tumors to systemic chemotherapy, which offers modest clinical benefits in improving overall survival [2]. Epidemiologic studies have connected several modifiable risk factors to the development and progression of this disease, including smoking, chronic alcohol consumption, and morbid obesity, all of which represent a significant public health burden nationwide [3]. Recent advances into the study of these risk factors have shown that they promote PDAC tumor growth through modulation of fundamentally distinct cytokine and kinase pathways [4]. Our knowledge of these mechanisms is rudimentary, but is rapidly evolving as the importance of these risk factors in PDAC becomes more evident. In an attempt to target convergent mediators in these processes, our group and others have recently identified cyclic AMP response element binding protein (CREB) as a key transcription factor involved in smoking, alcohol, and obesity-induced tumor initiation and progression and a potential target for the treatment of PDAC associated with these risk factor exposures (Figure 1).

Cigarette smoking has been extensively described as a major risk factor in the development of numerous solid organ malignancies including pancreatic cancer. Our findings show that exposure to a nicotine-derived nitrosamine, 4-(methylnitrosamino)-1-(3-pyridyl)-1-butanone (NNK), significantly accelerates pancreatic precancerous lesion (PanIN) formation in a genetically engineered mouse model of PDAC (*Ptf1a*^*CreERTM*^*; Kras*^*G12D/+*^*)* [5]. Furthermore, we have shown that chronic NNK exposure significantly enhances tumor growth both *in vitro* and *in vivo*, highlighting the significant role of smoking in both tumor formation and progression. In addition to direct effects on PDAC tumor cells, NNK exposure also exerts significant changes in the tumor microenvironment (TME). This includes enhanced infiltration of immunosuppressive cell populations into the TME, including regulatory T-cells (Tregs) and tumor-associated macrophages (TAMs). Additionally, the progression of stromal fibrosis, a major contributor of therapeutic resistance in PDAC, is also heavily influenced by smoking carcinogens, as NNK exposure *in vivo* results in a marked increase in tumor desmoplasia. Through mechanistic studies, we have demonstrated that the oncogenic effects of NNK exposure are mediated through the activation of CREB through downstream stimulation of the AKT/PI3K pathway. Moreover, inhibition of CREB has shown both direct and indirect influences on PDAC tumor growth, wherein it has directly impeded NNK-induced tumor cell proliferation and indirectly reduced immunosuppressive cell infiltration into TME and stromal fibrosis in PDAC [5].

Chronic alcohol consumption is implicated in the pathogenesis of multiple pancreatic disorders through the induction of acinar cell injury. Chronic alcohol exposure prompts a profound inflammatory response through repeated cellular damage in the pancreas, eventually leading to neoplastic transformation and the subsequent development of PDAC. Recent reports have shown that alcohol exerts its pro-tumorigenic effects by initiating a cellular reprogramming event known as acinar-to-ductal metaplasia, eventually advancing to PanIN formation and invasive cancer *in vivo* through activation of the AKT/PI3K pathway [6]. We have previously reported CREB as a downstream regulator of the AKT/PI3K signaling pathway that appears to be essential in mediating this malignant transformation. Our ongoing findings demonstrate that alcohol not only affects signaling pathways within PDAC tumor cells, but also stimulates CXCL12 release (Figure 1). CXCL12, also known as stromal cell-derived factor 1 (SDF1), is known to induce extensive stromal fibrosis through its interaction with CXCR4 and is key in the induction of multiple resistance mechanisms in cancer. It has been suggested that CXCL12 enhances cell survival synergistically with other cytokines and involves activation of CREB [7]. Furthermore, CXCL12 is known to be a chemotactic molecule which may affect the immune response in PDAC tumors, as previous studies have demonstrated that CXCL12 blockade may enhance the anti-tumor immune response through inhibition of MDSCs and Treg populations in other solid organ tumors [8]. Therefore, we speculate that targeting of the CXCR4/CREB axis is a promising avenue to improve the antitumor response in PDAC.

Obesity is another well-established risk factor in PDAC development. Obesity is known to induce a pro-inflammatory state that differentially regulates pro-oncogenic cytokines and growth factors that are key mediators of PDAC progression. We have previously reported that the IL-6/STAT3 pathway is the critical regulator of obesity-induced PDAC growth [9]. There is accumulating evidence suggesting the presence of CRE binding regions in the promoter region of IL-6, wherein activated CREB would lead to STAT3 activation through enhanced IL-6 transcription [10] (Figure 1). The IL-6/JAK/STAT3 axis is known to mediate multiple deleterious processes in cancer progression, including chemoresistance, invasion/metastasis, and profound host immunosuppression. These data suggest CREB as a targetable mediator involved in STAT3 signaling and present exciting new opportunities in the treatment of obesity-induced PDAC.

The association among multiple modifiable risk factors on the initiation and progression of PDAC is well described; however, our understanding of the diverse cellular mechanisms that are affected by these pathologic states remains unclear. It is apparent that smoking, alcohol exposure, and morbid obesity induce oncogenic effects through direct interaction with PDAC cells, but additionally they seem to exert profound effects on the immune cell population and architecture of the microenvironment that may mediate disease progression and resistance to therapy. Our studies have provided fundamental insights into PDAC carcinogenesis induced by these risk factors and suggest CREB as a key oncogenic driver and therapeutic target in these processes. Further investigations into the central role of CREB in risk factor-associated PDAC are needed to promote our understanding and expand the treatment options available for this deadly malignancy.
